# Comparative Analysis of CNN and RNN for Voice Pathology Detection

**DOI:** 10.1155/2021/6635964

**Published:** 2021-04-14

**Authors:** Sidra Abid Syed, Munaf Rashid, Samreen Hussain, Hira Zahid

**Affiliations:** ^1^Department of Biomedical Engineering and Department of Electrical Engineering, Ziauddin University Faculty of Engineering Science, Technology, and Management, Karachi, Pakistan; ^2^Department of Electrical Engineering and Department of Software Engineering, Ziauddin University Faculty of Engineering Science, Technology, and Management, Karachi, Pakistan; ^3^Vice Chancellor, Begum Nusrat Bhutto Women University, Sukkur, Pakistan; ^4^Department of Biomedical Engineering, Ziauddin University Faculty of Engineering Science, Technology, and Management, Karachi, Pakistan

## Abstract

Diagnosis on the basis of a computerized acoustic examination may play an incredibly important role in early diagnosis and in monitoring and even improving effective pathological speech diagnostics. Various acoustic metrics test the health of the voice. The precision of these parameters also has to do with algorithms for the detection of speech noise. The idea is to detect the disease pathology from the voice. First, we apply the feature extraction on the SVD dataset. After the feature extraction, the system input goes into the 27 neuronal layer neural networks that are convolutional and recurrent neural network. We divided the dataset into training and testing, and after 10 k-fold validation, the reported accuracies of CNN and RNN are 87.11% and 86.52%, respectively. A 10-fold cross-validation is used to evaluate the performance of the classifier. On a Linux workstation with one NVidia Titan X GPU, program code was written in Python using the TensorFlow package.

## 1. Introduction

Speech is one of the basic human instincts and voices of the subsystem. Natural voice is the auditory result of pulmonary air bursts communicating with the larynx, which sets the adduction of true vocal folds and creates intermittent and/or aperiodic sounds. Sometimes, numerous abusive vocal patterns, typically referred to as vocal hyperfunction, result in speech disorders such as aphonia (complete lack of voice and/or dysphonia (partial loss of voice) [1]). Speech dysfunction is something that deviates “quality, pitch, loudness, and/or vocal flexibility” from voices of common age, gender, and social classes [2]. The consequence of nonmalignant speech disorders is not life-threatening, but the effects of untreated voice dysfunction may have a major impact on social, occupational, and personal aspects of communication [3]. Of the numerous vocal fold lesions, mass pathologies are particularly prevalent due to the phonotraumatic effect on vulnerable multilayer vocal folds, persistent tissue infection, and environmental stimuli frequently resulting in vocal nodules and vocal polyps [4]. In these conditions, the closing of the vocal fold is insufficient, and the production of the voice is not economical and perceptually hoarse. In the opposite, there are no vocal fold lesions in nonphonotraumatic voice disorders, such as muscle tension dysphonia and functional speech dysfunction, but vocal exhaustion, degraded voice quality, and increased laryngeal discomfort may be found. Multiparametric evaluation methodology is known to be suitable for voice assessment [1, 5]. Historically, a systematic approach is important and includes the following: patient interview, laryngeal examination via stroboscopy and/or laryngoscopy, simple aerodynamic assessment, auditory analysis by standardized psychoacoustic approaches, auditory analysis, and subjective speech assessment. In view of recent technical advances, voice scientists have been at the forefront of the creation of acoustic processing instruments to discern natural voice from those with aphonia and/or dysphonia. Structures for the expression recognition of voice disorders can be planned and built utilizing machine learning (ML) algorithms. Here, the voice data must be preprocessed and transformed into a series of features before an ML algorithm is used [6]. Experts could manually mark a collection of speech data in audio files as a safe or defective expression. Then, the original audio data in each file is split into short frames, and each frame is analyzed to remove the features from it. The set of features derived from all frames is called feedback for neural networks. The data collection is split into training and research sets by randomly choosing observations of both natural and pathological voices. The training set is used to build the machine learning algorithm, and the test set is used to validate the model. The precision of the designation is determined during the assessment process. This precision of classification shall be taken as a metric for determining the efficiency of the different Automatic Voice Disorder Detection (AVDD) programs [7].

There are a few gaps identified by Abid Syed et al. [8] in the area of voice disorder detection through Artificial Intelligence techniques like the lack of using unsupervised techniques by researchers in the detection of voice orders, the lack of the accuracy comparison, or the less work on Arabic Voice Pathology Database (AVPD) [9]. In this paper, we have used Saarbruecken Voice Database (SVD) [10] for the detection of voice order. The proposed paper is the continuation of the previous work of the authors [11] in which they first applied Support Vector Machine (SVM), Decision Tree, Naïve Bayes, and Ensemble, and then on the same set of features and disease, Syed et al. proposed comparative analysis of RNN and CNN. The aim of this paper is to design a system by first extracting features and then applying recurrent neural network (RNN) as a machine learning classifier to predict the accuracy of the system. Secondly, we will compare the results of RNN with convolutional neural network (CNN) and also try to increase the reported accuracy of the system using CNN because previously the highest reported accuracy using convolutional neural network is 80% in the meta-analysis [8]. In this paper, we will be using the SVD dataset which has voice recordings of vowel sounds of the patient with the different disease.

## 2. Related Work

Al-Nasheri et al. in [12–14] used SVM on SVD [10] to propose a system for voice disorder detection. In [12], Al-Nasheri et al. focus on creating a reliable and robust function extraction to identify and distinguish voice pathologies by analyzing various frequency bands using autocorrelation and entropy. Maximum peak values and their related lag values were derived from each frame of the spoken signal using autocorrelation as a function to identify and distinguish pathological samples. We have obtained the entropy for each frame of the speech signal after we normalized the values to be used as functions. These features were examined in different frequency bands to determine the contribution of each band to the identification and classification systems. Various examples of continuous vocal for both natural and abnormal voices were collected from three separate datasets in English, German, and Arabic. The help vector machine has been used as a classifier. The highest reported accuracy is 92% for SVD. In [13], the main purpose of this paper is to analyze Multidimensional Voice Software (MDPV) parameters in order to automatically identify and distinguish voice pathologies in different datasets and then to figure out which parameters behaved well in these two processes. The experimental findings reveal a clear difference in the efficiency of the MDPV parameters utilizing these databases. Highly rated parameters often varied from one database to the next. The best accuracy was achieved by utilizing the three top rated MDVP metrics organized according to the Fisher Discrimination Ratio of 99.98% for SVD. In this article [14]; we derived maximal peak values and their related lag values from each frame of the spoken signal using the correlation method as a feature to identify and identify pathology materials. These characteristics are studied in various frequency bands to see the contribution of each band to the identification and classification processes. The most contributive bands for both identification and designation are between the 1000 and 8000 Hz. The maximum rate of precision gained by utilizing cross-correlation is 99.809%, 90.979%, and 91.16% in the Massachusetts Eye and Ear Infirmary, Saarbruecken Speech Database (SVD), and the Arabic Voice Pathology Database, respectively. However, the maximum rate of precision acquired by utilizing cross-correlation was 99.255%, 98.941%, and 95.188%, respectively, in the three datasets. In [15, 16], Teixeira et al. proposed the system for voice detection keeping the same features in both of his publication but changing the classifiers. In [15], they used SVM with Jitter, shimmer, and HNR and the reported accuracy was 71%. In [16], they used MLP-ANN with Jitter, shimmer, and HNR and the reported accuracy was 100% but only for female voices. In [17], Fonseca et al. used SVM with SE, ZCRs, and SH and the reported accuracy was 95%.

Also, there is not much work done for voice pathology using a convolutional neural network. Only Guedes et al. [18] designed a system and reported an accuracy of 80%, and Zhang et al. [19] also use the DNN model which was machine learning where outcomes were missing. So after a detailed literature review, it was concluded that a novel system can be proposed using pitch, 13 MFCC, rolloff, ZCR, energy entropy, spectral flux, spectral centroid, and energy as features and RNN as a classifier to increase the accuracy and further using CNN to verified the results.

## 3. Materials and Method

### 3.1. Dataset

SVD stands for Saarbrücken Voice Database. In Table 1, the characteristics of SVD dataset are presented. Basically, SVD is a publically available database which is a collection of voice recordings by over 2000 people with over 72 voice pathological conditions: (1) vocal registration [I a, u] produced at standard, high, and low pitches, in which the truth was recorded in a recording session;(2) vocal documentation of increasing pitch [I a, u]; and (3) recording of the phrase “Good morning, how do you like it?” (“How are you, good morning?”). The voice signal and the EGG signal were stored in individual files for the specified components [11]. The database has text file including all relevant information about the dataset. Those characteristics make it a good choice for experimenters to use. All recorded SVD voices were sampled with a resolution of 16-bit at 50 kHz. There are some recording sessions where not all vowels are included in each version, depending on the quality of their recording. The “Saarbruecken Voice Server” is available via this web interface. It contains multiple internet pages which are used to choose parameters for the database application, to play directly and record and pick the recording session files which are to be exported after choosing the desired parameter from the SVD database [12]. From the SVD database, the disease we have selected are “Balbuties,” “Dysphonie,” “Frontolaterale Teilresektion,” “Funktionelle Dysphonie,” “Vox senilis,” “Zentral-laryngaleBewegungsstörung,” “ReinkeÖdem,” “Stimmlippenpolyp,” “Stimmlippenkarzinom,” “SpasmodischeDysphonie,” “Psychogene Dysphonie,” and “Leukoplakie” [11]. The diseases were solely selected on the basis of common diagnosis of voice disorders.

### 3.2. Feature

The features that are extracted from samples to perform this study are 13 MFCC features, pitch, rolloff, ZCR, energy entropy, spectral flux, spectral centroid, and energy. Syed et al. in their previous work [11] add seven more features, i.e., pitch, rolloff, ZCR, energy entropy, spectral flux, spectral centroid, and energy, to produce more enhanced voice sample for processing.

#### 3.2.1. Mel-Frequency Cepstral Coefficients (MFCC)

In 1980, MFCC was suggested by Davis and Mermelstein for the most widely used speech recognition feature [20]. Primarily, the exhaustion method for the MFCC function involves windowing the signal, applying the DFT, acquiring the magnitude protocol, and then shaming the values and a Mel rank on scale, then applying a reverse DCT. The cepstral coefficients normally include only details from a specific frame and are considered static attributes. The machine first and second derivatives of cepstral coefficients have the additional information on time dynamics of the signal [21].

(1) $$\begin{array}{c} {\hat{y}}_{t}\left[j\right]=\frac{y_{t}\left[j\right]-\mu \left(y\left[j\right]\right)}{\sigma \left(y\left[j\right]\right)}. \end{array}$$

#### 3.2.2. Pitch

The pitch corresponds to the level at which during a noise voicing cord vibrates. Standard approaches such as the autocorrelation system and the method of average magnitude differential at max, resulting in half and double-half defects, are vulnerable to mutation during the removal of tonnes. By distinguishing the acoustic pulse cepstrum from the vocal tract cepstrum, the cepstrum system may approximate the pitch. At the cost of complex measurements, it has high detection performance for regular voice signal [19].

### 3.3. Neural Networks

#### 3.3.1. CNN Architecture

The CNN has several hierarchy levels composed of routing layers and grouping layers, which are defined by a broad variety of charts. In general, CNN begins with a convolutionary layer that accepts input level data. For convolutionary operations with few filter maps of the same dimension, the convolution layer is liable. In addition, the output from this layer is transferred to the sample layer that decreases the scale of the next layers. CNN is locally related to a vast variety of deep learning techniques. These networks are then implemented on the basis of GPU architecture on a number of hundred cores. The role maps will be allocated on the basis of the previous layer knowledge blocks [22]. It depends on the dimensions of the maps. However, each thread is bound to a single neuron by means of a single block of many threads. Similarly, neuron convolution, induction, and summation are carried out over the remainder of the method. Finally, a global memory stores the performance of the above processes. A backward and propagation model is adopted for the efficient processing of results. However, a single spread would not yield positive outcomes, so pulling or moving operations contribute to parallel spread. In addition, the neurons of a single layer interact with a separate number of neurons, influencing the boundary effects [23].

**Figure 1:**
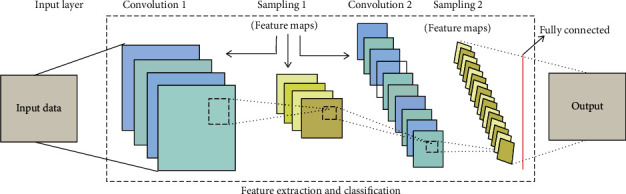
Architecture of CNN [22].

In Figure 1, the general architecture of CNN is explaining the work of this deep learning neural network. A deep learning algorithm includes input preprocessing, deep learning model training, storage of the learned model, and the last phase of the model implementation. In these phases, the most computational (or data intensive activity is to train the deep learning algorithms (defining and running). The model is provided some input through a neural network that produces some output at the specified step (also called forward transmission). The weights are changed if the performance is inappropriate or inaccurate (backward pass). This could be like a basic matrix multiplication, where input (first matrix row) for such unique output objects is multiplied by weight (second matrix column). Serial systems (CPU-based) are typically not feasible for higher order matrices (large inputs and weighs). Fortunately, GPU delivers much superior options than conventional single or cluster CPU systems [24] of graphic processing units for general purposes.

#### 3.3.2. RNN Architecture

**Figure 2:**
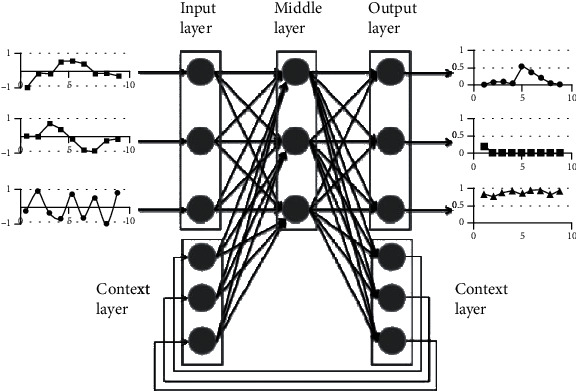
Architecture of LSTM-RNN [25].

Long short-Term Memory (LSTM) is a special architecture of the recurring neural network (RNN) constructed more reliably than traditional RNNs and is designed to model temporal sequences and their long-range dependencies. Recently, we have shown that LSTM-RNN is more powerful than DNNs and standard acoustic modelling, taking into account models of moderate size trained on a single computer. We illustrate the potential to achieve the newest technology in speech recognition with a two-layer deep LSTM-RNN with a linear repeating projection layer. In Figure 2, the LSTM-RNN general architecture represents the working flow of the model. This design uses the model parameters more efficiently than other parameters, converges fast, and outperforms a deep neural network feed with a higher magnitude order. Speaking is a dynamic signal with time fluctuations with complex associations on a number of timescales. Recurring neural networks (RNs) have cyclic ties that render them more efficient than feedforward neural networks in modelling certain sequence data. RNNs have been very effective in sequence marking and prediction activities such as handwriting and language detection [25].

The key distinction between CNN and RNN is the capacity to process transient or sequentially produced knowledge for example, in a phrase. In comparison, convolutionary neural networks and repetitive neural networks are used for entirely different uses, and the neural network architectures themselves vary to match these different cases of use. In order to convert results, CNNs use filter in convolution layers. In comparison, RNNs reuse activation functions from other sequential data points to build the following sequence production. Although this is an often discussed query, the distinction between CNN and RNN becomes apparent as you analyze the nature of neural networks and realize what they are used for.

## 4. Experiments and Evaluation

### 4.1. Proposed Model Layer of CNN and RNN

At the beginning of any study, the data needs cleaning, organized and error free. For any dataset loaded into the Python Pandas DataFrame, it is almost always necessary to remove different rows and columns in order to get the correct data collection for your particular study or visualization. In python for a simplified data model, we have used the command of “DataFrame.drop ()” which drop all the unnecessary columns and frames and give the simplified version of the speech samples. By defining label names and related axes or by explicitly specifying index or column names, you may exclude rows or columns. Labels from various levels may be eliminated by using a multiindex by defining the rank [26].

Similarly, for preprocessing, we have import “train_test_split” from “sklearn.model_selection” and “LabelEncoder” and “StandardScaler” from “sklear.preprocessing” The train-test split protocol is used to approximate the accuracy of machine learning algorithms as they are used to make decisions about data not used to train the model. It is a short and simple process to run, the results of which enable you to compare the output of machine learning algorithms with your predictive modeling problem. While it is easy to use and interpret, there are occasions where the protocol may not be utilized, such as when you have a limited dataset and cases when extra tuning is needed, such as when it is used for classification and when the dataset is not balanced. The technique entails the acquisition of a dataset and the division into two subsets. The first subset is used to match the model and is called the testing dataset. The second subset is not used to train the model; however, the dataset input element is given to the computer; then the predictions are rendered and compared to the predicted values. The second dataset is referred to the test dataset [27]. The 2000 selected recordings from the dataset were randomly divided into 80% and 20%. The 80% was included for training, and 20% was included was testing.

**Figure 3:**
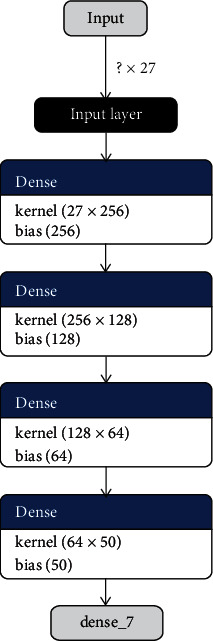
Proposed in-layer model of CNN.

**Figure 4:**
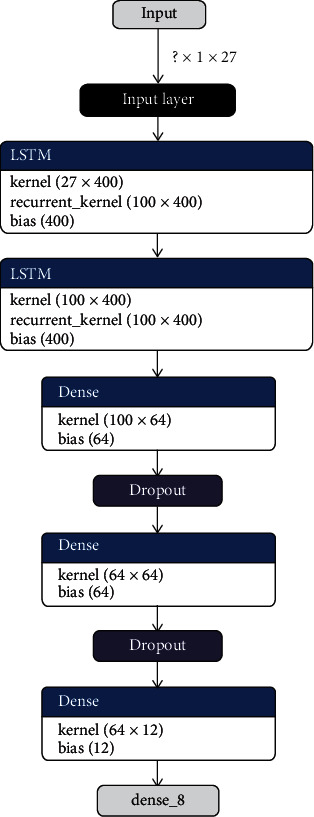
Proposed in-layer model of LSTM-RNN.

Figures 3 and 4 demonstrate the internal layering diagram of the proposed model of CNN and RNN. In proposed methodology, both CNN and RNN are 27 neuronal layer architectures with different bias values.

### 4.2. Results

**Figure 5:**
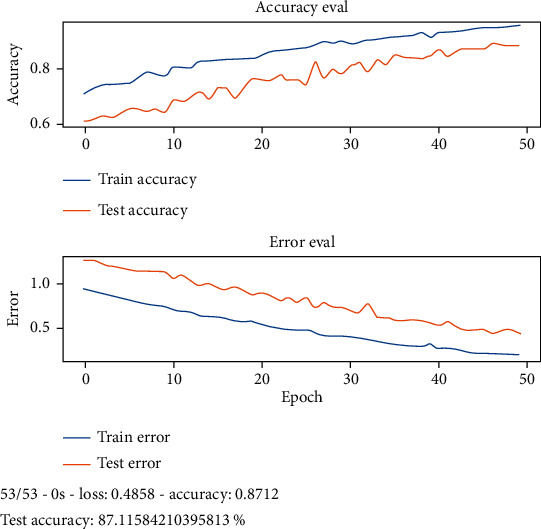
Accuracy and error evaluation of CNN in training and testing phase.

**Figure 6:**
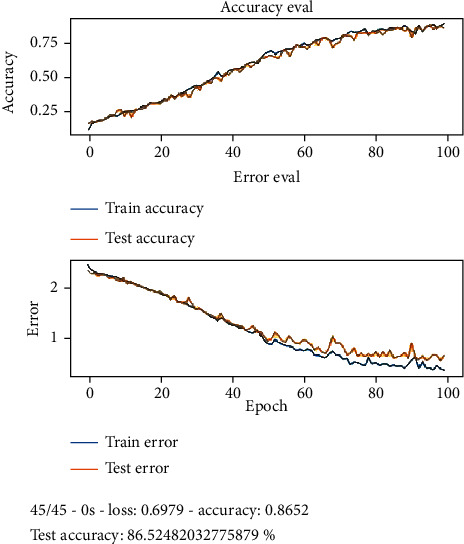
Accuracy and error evaluation of RNN in training and testing phase.

**Figure 7:**
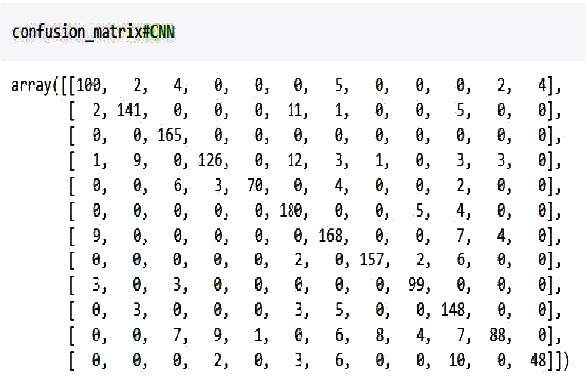
Confusion matrix of CNN.

**Figure 8:**
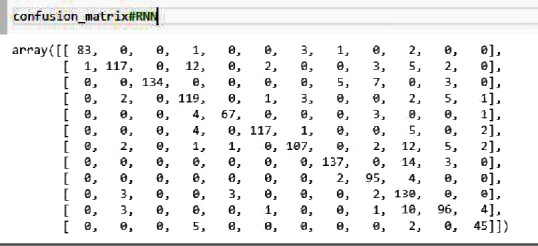
Confusion matrix of LSTM-RNN.

The idea is to detect the disease pathology from the voice. First, we apply the feature extraction on the SVD dataset. In proposed methodology, the features that we have extracted are 13 MFCC features, pitch, Rolloff, ZCR, energy entropy, spectral flux, spectral centroid, and energy. After the feature extraction, the system input goes into the 27 neuronal layer neural networks that are convolutional and recurrent neural network. We divided the dataset into training and testing, and after 10 k-fold validation, the reported accuracies of CNN and RNN in Table 2 are 87.11% and 86.52%, respectively. There were 7 residual layers of the convoluted kernels of 27 residual blocks. Dropout with a frequency of 0.5 was used to maintain L2 normalization. For success assessment, 10-fold cross-validation has been used. Software code has been published on a workstation with one NVidia Titan X GPU using the TensorFlow plugin in python. Figures 5 and 6 represent the detailed accuracy and error evaluation with the lines drawn for training testing phase. The graph lines are joined in RNN evaluations which shows that the error margin is very minor, but in CNN evaluation, there are differences between the lines which show the probability of error margin in the proposed CNN algorithm which is higher than the RNN. Figures 7 and 8 represent the confusion matrix with the value that shows the number of correct diagnosis of the system.

## 5. Limitation

We understand that our neural network classifiers always have to attain optimal results. The exact measures, however, are superior to or close to other reported NLP reports. For example, while 10 of the 1000 test cases in the CNN model were a mistake, the classification errors in neural networks are very challenging to explore, since they are mostly “black boxes”. A lack of direct mention of the primary cause of error was PE and limited documentation because of shortage or absence of insufficient quality of the image; inference based on the context was required instead. The model focused on RNN correctly forecasts the groups and located the most relevant sentences of the papers, but the model's inference is still difficult to generalize. We have seen just one case of a positive/negative PE classification, where CNN correctly forecasts it to be positive, but RNN forecasts it to be negative. It was not therefore evident how CNN might correctly predict this case on the basis of the heat map produced. Therefore, all of these mistakes need a subtle logic, which can restrict the design of our models, in addition to training limits raised by the scale of our data sets.

## 6. Conclusion

The amount of work done in this field concluded that clinical diagnosis voice disorders through machine learning algorithms have been the area of interest for most researchers. Hence, after applying the proposed methodology, we are able to increase the accuracy of the convolutional neural network which is 87.11% which is increased from the accuracy reported in the literature review. Comparatively, the accuracy of the recurrent neural network also closes to CNN and the predicted outcomes were almost the same. For future work, continuing to work with a neural network in the SVD dataset for the detection of voice pathology can report better accuracies.

## Figures and Tables

**Table 1:** Characteristics of SVD dataset.

Dataset	SVD		
Characteristics	Language	Sampling frequency	Text
	German	50 KHz	Vowel /a/
			(1) Vowel /i/
			(2) Vowel /u/
			(3) Sentence

**Table 2:** Accuracy of RNN and CNN at 10-fold verification.

Algorithm	Validation	Accuracy
CNN	10-fold	87.11%
LSTM-RNN	10-fold	86.52%

## Data Availability

The data base used for this particular study is an open source data available at this link http://www.stimmdatenbank.coli.uni-saarland.de/help_en.php4.
